# Draft Genome Sequence of the Wood-Decaying Fungus *Xylaria* sp. BCC 1067

**DOI:** 10.1128/MRA.00512-19

**Published:** 2019-06-20

**Authors:** Sawannee Sutheeworapong, Nuthatai Suteerapongpan, Prasobsook Paenkaew, Peerada Prommeenate, Supapon Cheevadhanarak, Songsak Wattanachaisaereekul

**Affiliations:** a Pilot Plant Development and Training Institute, King Mongkut’s University of Technology Thonburi, Bangkok, Thailand; b School of Bioresources and Technology, King Mongkut’s University of Technology Thonburi, Bangkok, Thailand; c Biochemical Engineering and Pilot Plant Research and Development Unit, National Center for Genetic Engineering and Biotechnology, National Science and Technology Development Agency at King Mongkut's University of Technology Thonburi, Thonburi, Bangkok, Thailand; Vanderbilt University

## Abstract

*Xylaria* sp. BCC 1067 is a wood-decaying fungus which is capable of producing lignocellulolytic enzymes. Based on the results of a single-molecule real-time sequencing technology analysis, we present the first draft genome of *Xylaria* sp. BCC 1067, comprising 54.1 Mb with 12,112 protein-coding genes.

## ANNOUNCEMENT

Lignocellulolytic enzymes are widely exploited in various applications, particularly in the hydrolysis stages of lignocellulose-based industries (1, 2). *Xylaria* species belonging to the class Sordariomycetes in the phylum Ascomycota are considered one of the most efficient types of wood-decaying fungi and are classified as soft-rot type II (3). *Xylaria* sp. BCC 1067, isolated from Nam Nao National Park in Thailand (4, 5), has been shown to produce lignocellulolytic enzymes, including endoglucanase, β-glucosidase, xylanase, and laccase. However, the genes underlying the production of enzymes from this fungus have never been explored and characterized. Therefore, the whole-genome *de novo* sequencing of *Xylaria* sp. BCC 1067 was performed in order to further understand the lignocellulolytic enzyme systems of this fungus.

Genomic DNA of *Xylaria* sp. BCC 1067 was extracted from mycelia cultivated in potato dextrose broth at 25°C for 5 days by the phenol-chloroform method (6). Genome sequencing was performed using PacBio single-molecule real-time (SMRT) sequencing technology. High-molecular-weight genomic DNA was sheared and selected on a BluePippin system using a cutoff range of 15 to 50 kb. The libraries were sequenced on 16 SMRT cells using a PacBio RS II sequencer according to the manufacturer’s instructions (Pacific Biosciences, Menlo Park, CA, USA). For *de novo* assembly, a total of 8,912,350,715 bases of genome sequences with 45,112 circular consensus sequence (CCS) reads were assembled using the SMRT Analysis Hierarchical Genome Assembly Process 3 (HGAP3) pipeline, which resulted in 43 contigs, with the length of the longest contig and the *N*_50_ value being 6,684,005 bp and 5,573,684 bp, respectively. The total size of the *Xylaria* sp. BCC 1067 genome was 54,100,337 bp, and the cumulative G+C content of the genome was 42.44%.

*Ab initio* gene prediction was carried out using Fgenesh v4.0.0 (7) based on gene models from Fusarium graminearum. As a result, 12,112 coding sequences (CDSs) were predicted. According to tRNAscan-SE v2.0 (8), the genome contained 232 nuclear tRNAs. CDSs were functionally characterized using BLASTP v2.2.31+ (9). A BLAST database was created from all protein sequences of 433 fungi in the phylum Ascomycota downloaded from the NCBI FTP site on 12 January 2017. The carbohydrate-active enzyme (CAZyme) gene content was determined using dbCAN2 (10, 11) based on three different tools, HMMER v3.2.1, DIAMOND v0.9.24, and Hotpep (12). The analysis revealed that the *Xylaria* sp. BCC 1067 genome contains 239 glycoside hydrolases (GH), 100 glycosyltransferases (GT), 16 polysaccharide lyases (PL), 27 carbohydrate esterases (CE), 87 carbohydrate-binding modules (CBM), and 86 enzymes with auxiliary activities (AA), indicating that this fungus has high potential to be used for biomass conversion. The genomic information of *Xylaria* sp. BCC 1067 will provide a better understanding of the lignocellulolytic enzyme system in this organism and could enable metabolic engineering of the strain for enhanced lignocellulolytic enzyme production.

### Data availability.

The draft genome sequence of *Xylaria* sp. BCC 1067 has been deposited in GenBank under the accession number SSCS00000000, BioProject number PRJNA531792, and BioSample number SAMN08619538. The raw reads have been deposited in the NCBI Sequence Read Archive (SRA) under the number SRP191752.
